# Supplementary material for: A monoclonal antibody that inhibits the shedding of CD16a and CD16b and promotes antibody-dependent cellular cytotoxicity against tumors
Source: Nat Commun. 2025 Nov 11;16:9915. doi: 10.1038/s41467-025-64862-5 (PMC12606087; doi:10.1038/s41467-025-64862-5)
Supplement: Supplementary file 1 — Supplementary Information [file 41467_2025_64862_MOESM1_ESM.pdf]

Supplementary Materials

A monoclonal antibody that inhibits the shedding of CD16a and CD16b and promotes antibody-dependent cellular cytotoxicity against tumors

Bruna Taciane da Silva Bortoleti and Lucas Ferrari de Andrade.

The PDF file includes:

Abbreviation list
Supplementary Figures 1 – 22, and their legends.
Supplementary Table 1

Abbreviation list
ADAM17 = A disintegrin and metalloprotease 17
ADCC = antibody-dependent cellular cytotoxicity
ADCP = antibody-dependent cellular phagocytosis
ANOVA = one-way analysis of variance
CD16a/b = CD16a and CD16b
CRC = colorectal cancer
D-7 = day minus seven (one week) before tumor cell inoculation
D10 = day ten after tumor cell inoculation
DANA = D265A and N297A
EC50 = half maximal effective concentration
EGFR = epidermal growth factor receptor
ELISA = enzyme-linked immunosorbent assay
Fab = fragment antigen binding
Fc = fragment crystallizable
GAALIE = G236A, A330L, and I332E
hEGFR = human epidermal growth factor receptor
HER2 = human epidermal growth factor receptor 2
hFcR = Fcγ receptor-humanized mice
hIgG1 = human IgG1
Ig = Immunoglobulin
IHC = Immunohistochemistry
iPSC = induced pluripotent stem cell
kDa = kilo Daltons
mAb = monoclonal antibody
MFI = mean fluorescence intensity
mIgG1 = murine IgG1
mIgG2a = murine IgG2a
NK cell = natural killer cell
NSCLC = non-small cell lung cancer
PE = phycoerythrin
PMA = phorbol myristate acetate
WT = Wild type

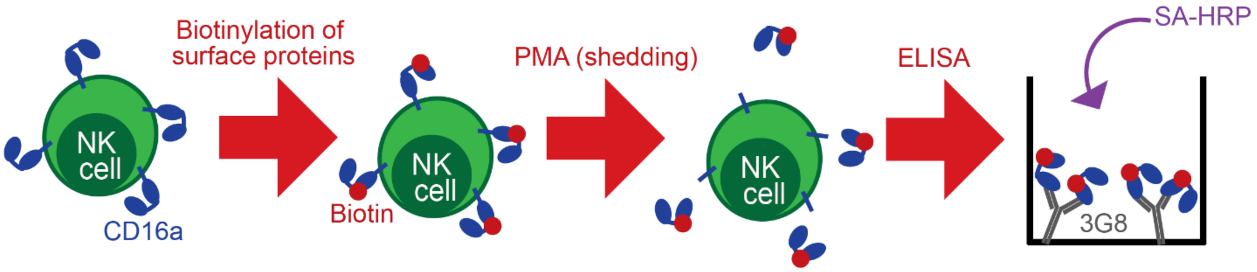

Supplementary Figure 1. Illustration of the assay for detection of soluble CD16a shed by NK cells. In this assay, primary NK cells have the surface proteins biotinylated and then immediately undergo PMA treatment. Hence, the surface proteins shed in supernatants are biotinylated. In this case, we focused on CD16a. Biotinylated CD16a is captured in ELISA plates with 3G8, a mAb that binds CD16a and CD16b. Peroxidase-labelled streptavidin (SA-HRP) is used to detect biotinylated CD16a (NK cells do not express CD16b). Figure 1B has data that was generated through this assay.

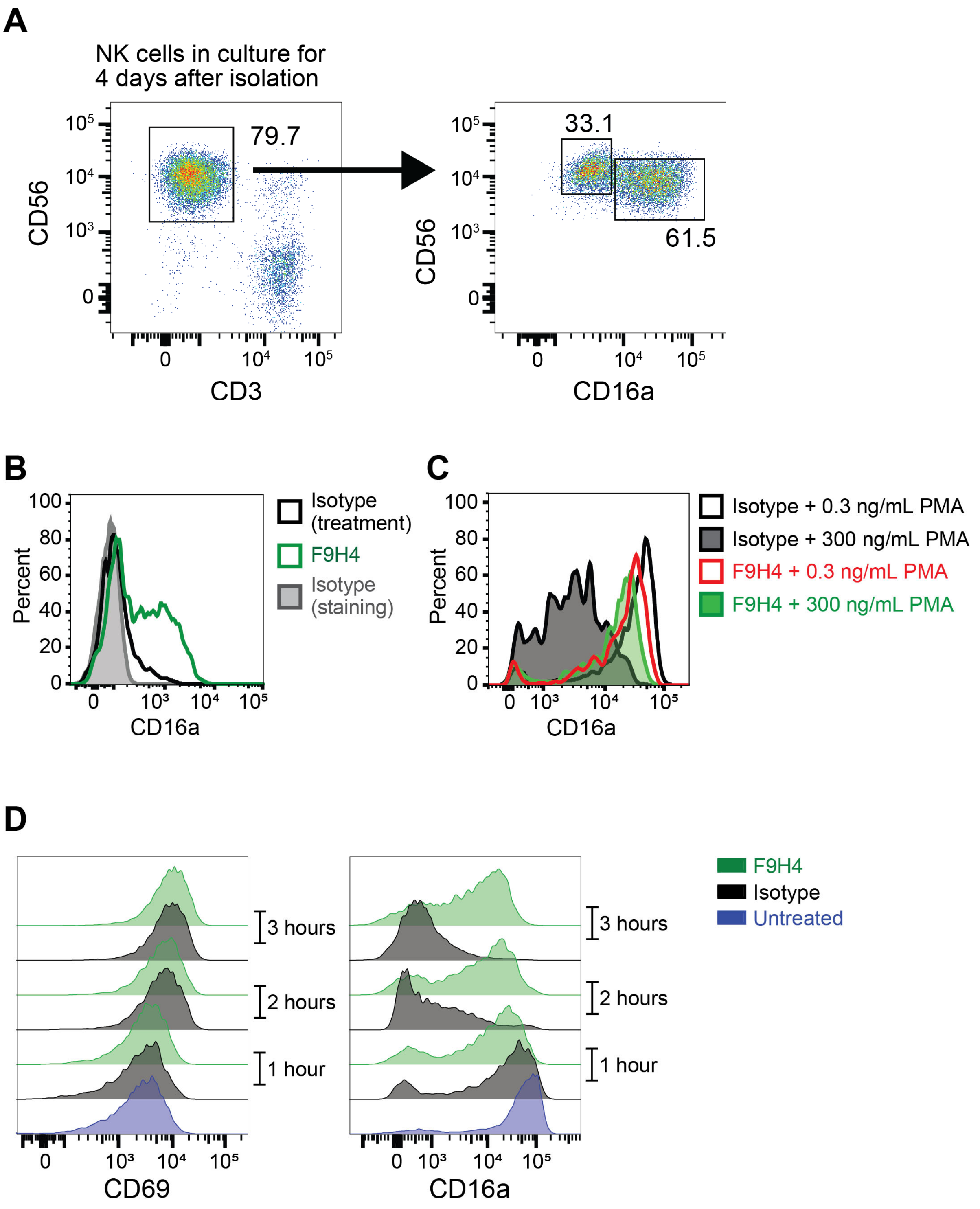

Supplementary Figure 2. F9H4 inhibits the CD16a shedding by human NK cells. (A) Example of validation of human NK cell purity after isolation by negative selection and four-day culture. **(B-D)** Flow cytometry histograms of data that represent the main Figures 1C (A), 1D (B), and 1E.

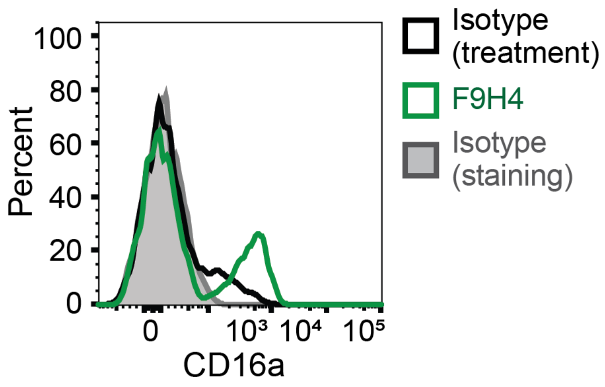

Supplementary Figure 3. F9H4 inhibits the CD16a shedding by human monocyte-derived macrophages. Data representing the experiment in the main Figure 1F, at the antibody dose of 10 μg/mL.

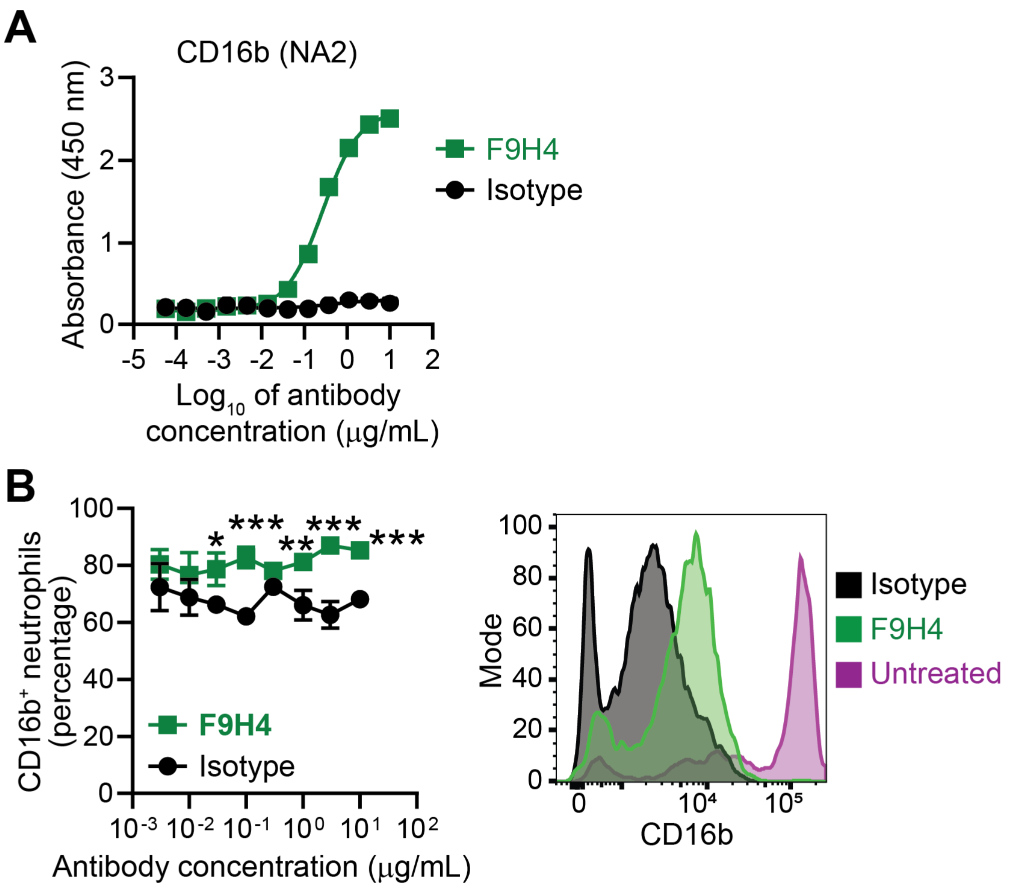

Supplementary Figure 4. F9H4 binds CD16b and inhibits the CD16b shedding by neutrophils. (A) ELISA for the reactivity of F9H4 to recombinant human CD16b protein. **(B)** CD16b-shedding assay with human primary neutrophils treated with PMA. Data represent three independent experiments (A-B), are mean +/- standard error of triplicates (B), and were analyzed by two-way ANOVA with Bonferroni's test (B). The histograms in B represent the CD16b expression levels in neutrophils with the antibodies at concentrations of 10 μg/mL.

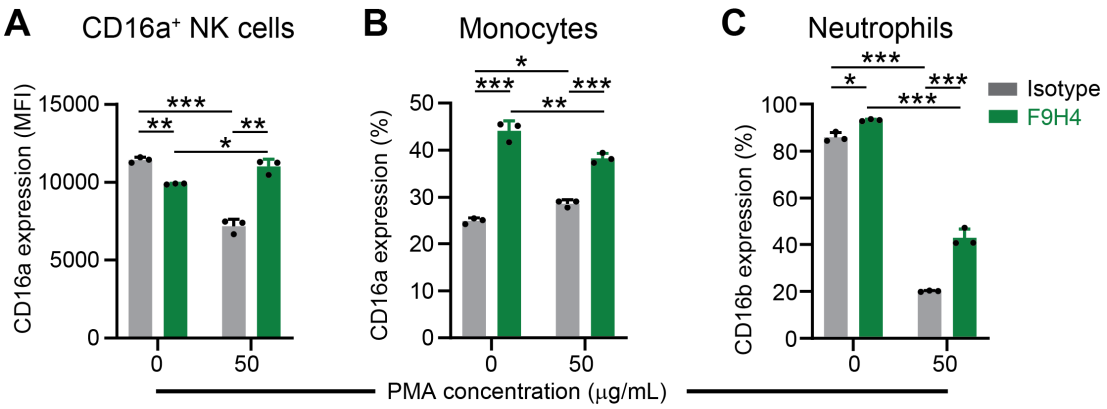

Supplementary Figure 5. CD16a/b shedding assay with fresh NK cells, monocytes, and neutrophils. Human PBMC were incubated for one hour with 50 ng/mL PMA or PBS plus F9H4 as mIgG1 or isotype control at dose of 10 μg/mL. Subsequently, surface CD16a/b expression levels were analyzed by flow cytometry. Monocytes (B) were identified with the CD45 and CD11b markers, neutrophils (C) were identified with the CD45 and CD15 markers, and NK cells (A) were identified based on lymphocyte gating with FSC-A and SSC-A, CD45 expression, and negative expressions of CD11b and CD15. Data are mean ± standard deviation of triplicates, represent three independent experiments, and were analyzed by two-way ANOVA with Bonferroni's test. *p<0.05, **p<0.01, and ***p<0.001.

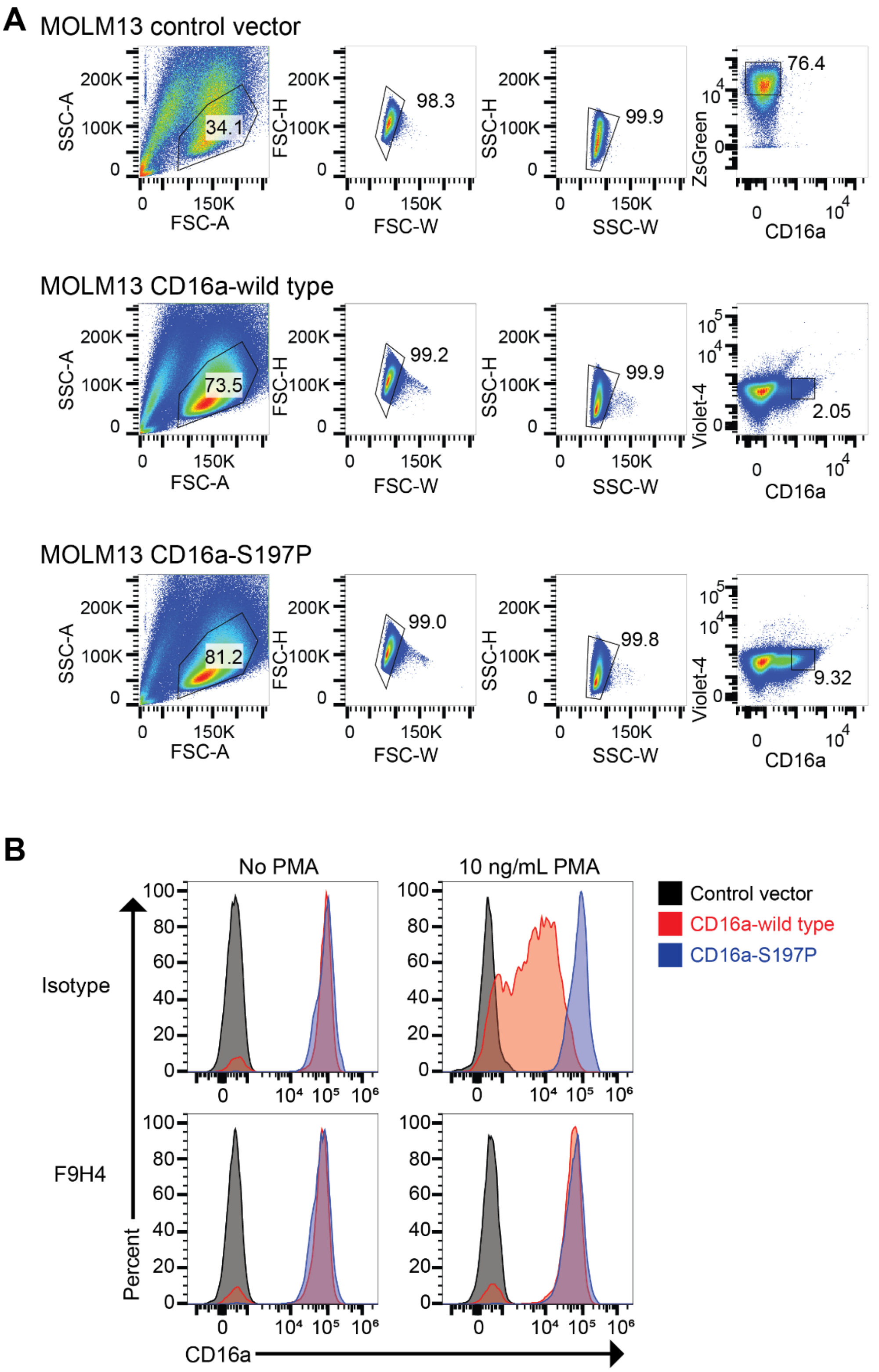

Supplementary Figure 6. Sorting of MOLM13 and demonstration that F9H4 inhibits the CD16a shedding by MOLM13-CD16a^{WT}. (A) Gating strategy for the sorting of MOLM13 after lentivirus treatment. **(B)** Histograms that represent the data in main Figure 1H.

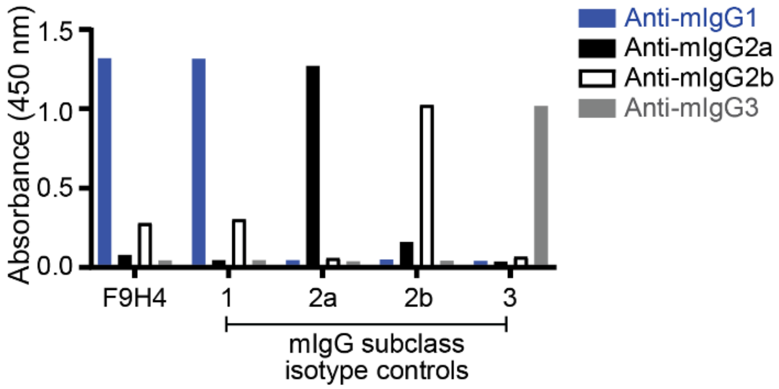

Supplementary Figure 7. F9H4 is mouse IgG1 (mIgG1). F9H4 and the indicated isotype controls were immobilized in multi-well ELISA plates, with one antibody type per well. Subsequently, the wells were incubated with the indicated secondary antibodies that were biotinylated, peroxidase-labelled streptavidin, TMB, and sulfuric acid. Plates were analyzed in microplate reader. Data are one replicate per bar and represent three independent experiments.

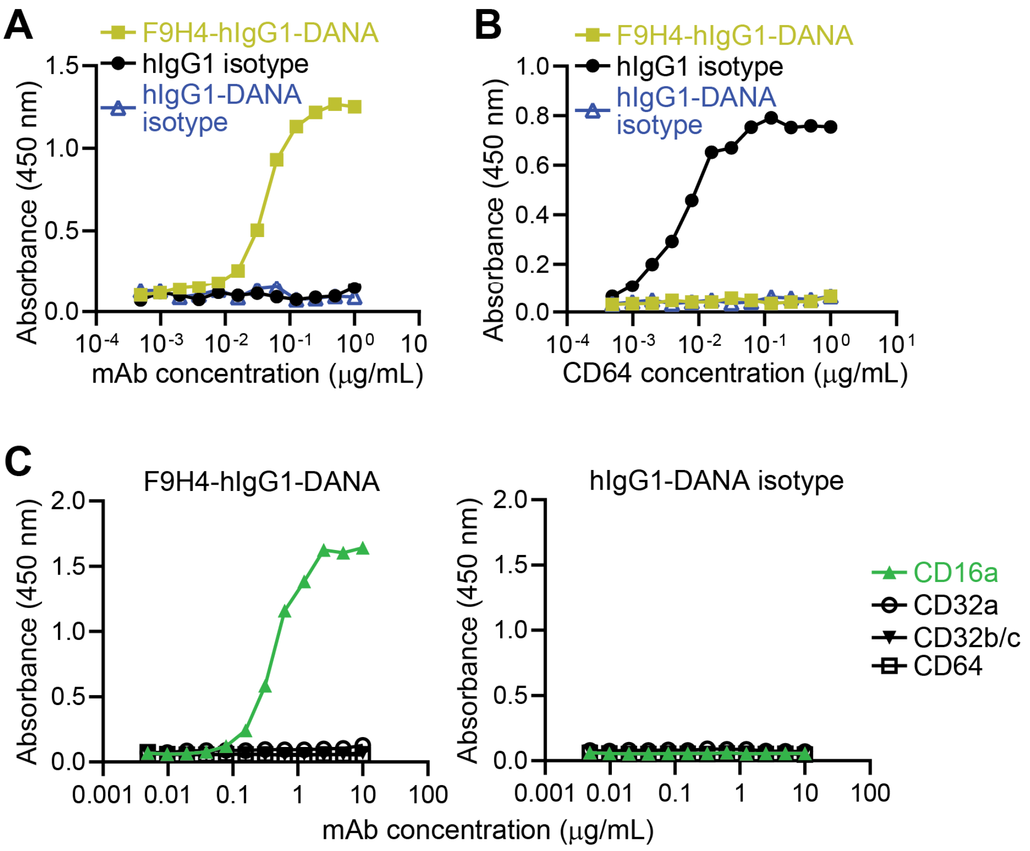

Supplementary Figure 8. Validation of the DANA-mutant humanized version of F9H4. (A) ELISA for reactivity of antibodies against human CD16a. **(B)** The indicated antibodies were immobilized in ELISA plates and detected by biotinylated human CD64 protein, which was added in the indicated concentrations. **(C)** ELISA for the binding of F9H4-DANA or isotype control to the indicated recombinant human Fc gamma receptors. Data are one replicate per data point and represent three independent experiments (A-C).

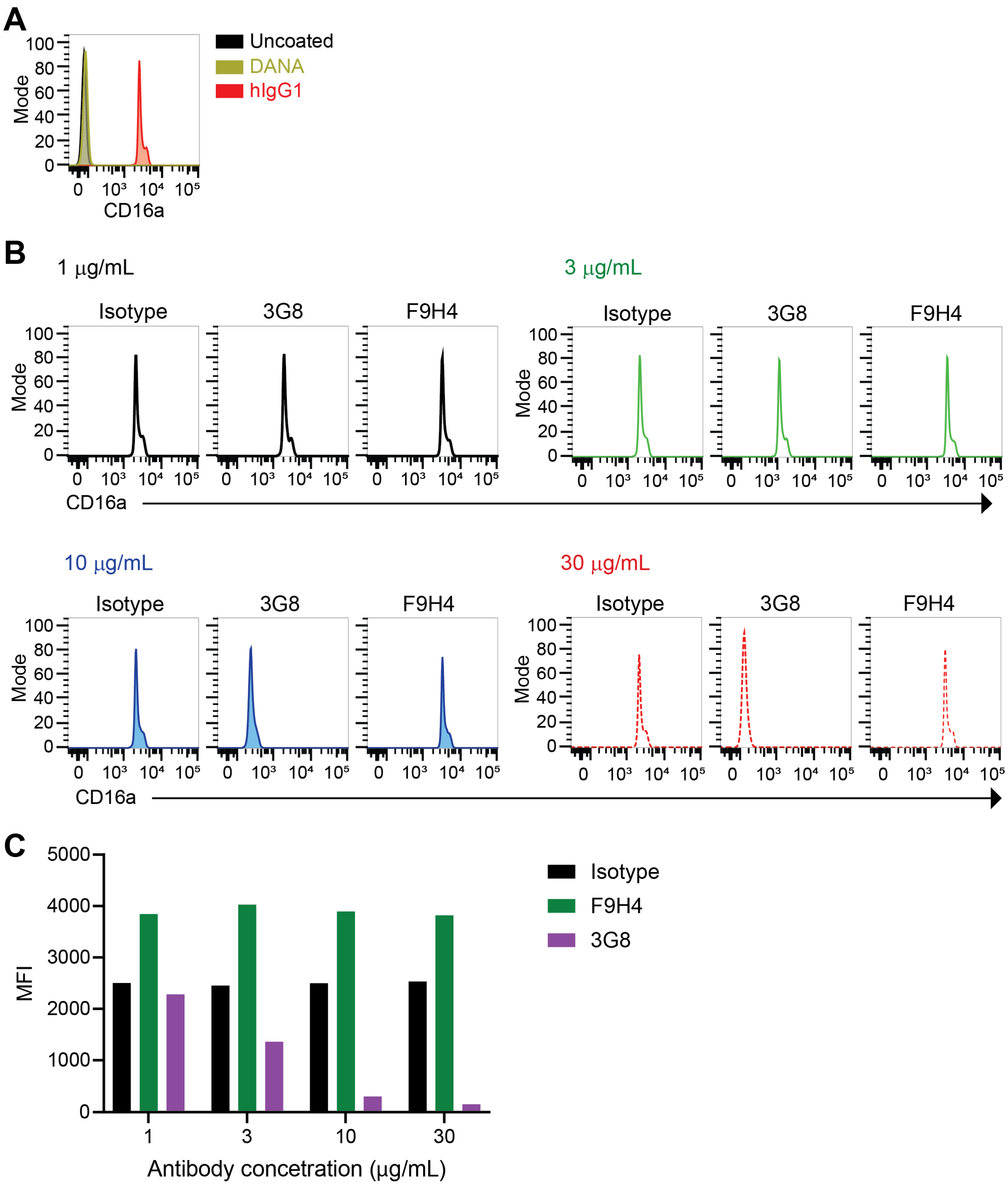

Supplementary Figure 9. The CD16a-hIgG1 binding assay by flow cytometry. Flow cytometry beads were chemically conjugated to hIgG1 wild type or hIgG1-DANA, followed by incubation with biotinylated CD16a, PE-labelled streptavidin, and flow cytometry analyses. **(A)** Validation of the assay. **(B)** Histogram deconvolution of the data shown in Figure 2B. Data represent three independent experiments (A-B).

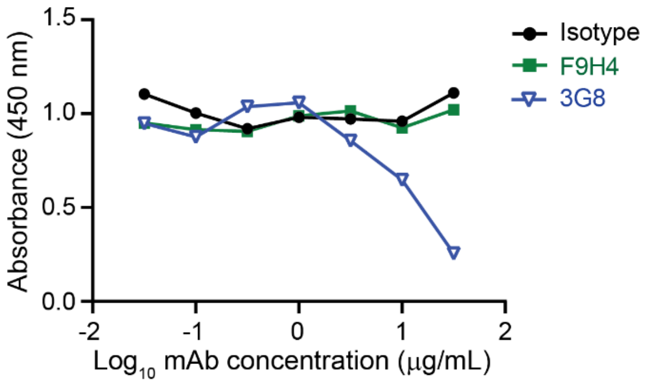

Supplementary Figure 10. F9H4 and 3G8 did not compete against each other to bind CD16a. Recombinant human CD16a protein was immobilized in multi-well ELISA plates, followed by the co-incubation of biotinylated 3G8 (1 μg/mL) with the indicated antibodies. Unconjugated 3G8 served as the positive control, because it competes against biotinylated 3G8. Data are one replicate per antibody dose and represent three independent experiments.

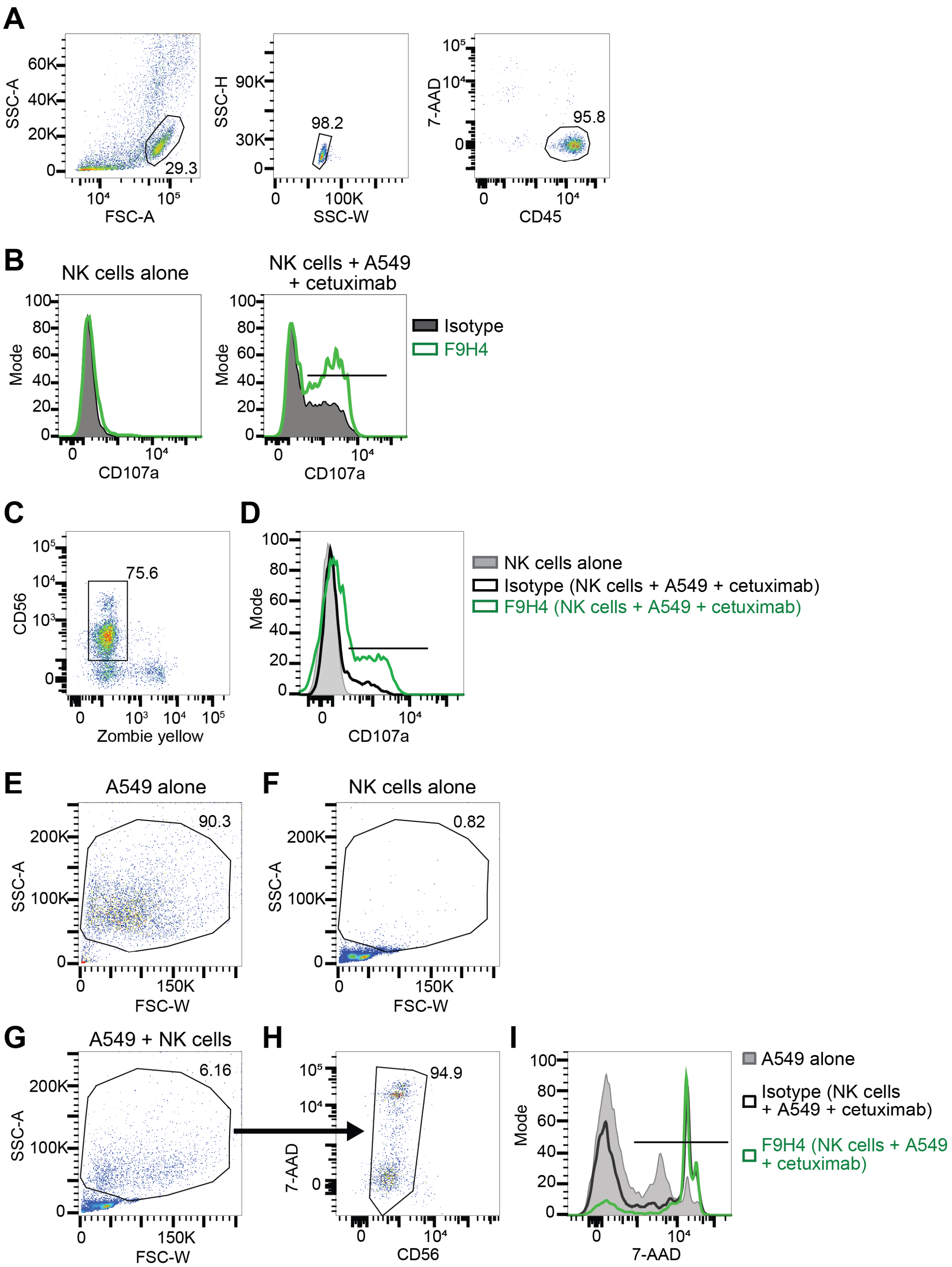

Supplementary Figure 11. Flow cytometry data that represent the data in Figure 3A-C. (A-B) Gating strategy for differentiation of NK cells from tumor cells for the main data in Figure 3A (A), and representative histograms for data in Figure 3A (B). **(C-D)** Illustration of NK cell

identification and representative histograms for the data in Figure 3B. **(E-I)** The flow cytometry-based cytotoxicity assay that was used in Figure 3A (E-I), and the data representing Figure 3C (I). The horizontal bar indicates dead cells, which were positive for 7-AAD (I).

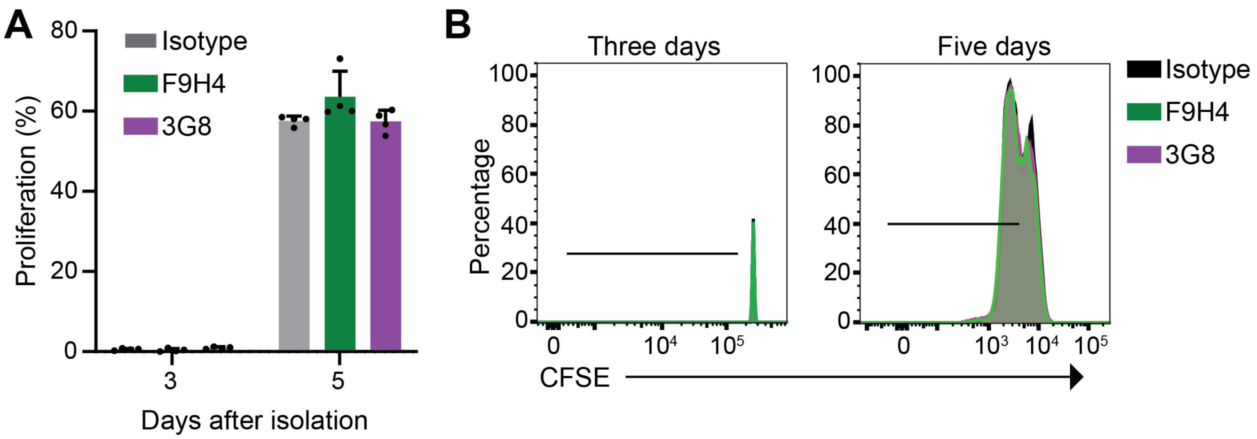

Supplementary Figure 12. F9H4 did not induce NK cell proliferation. (A-B) Blood NK cells were isolated by negative selection from the buffy coats of volunteer donors and cultured for seven days. Then, NK cells were labelled with CFSE and treated with the indicated mAbs for another three or five days, followed by analyses by flow cytometry. Data are mean ± standard deviation of four replicates (A) and represent three independent experiments (A). The histograms in B represent one of the observations per antibody group in A. The horizontal bars indicate cell proliferation (B).

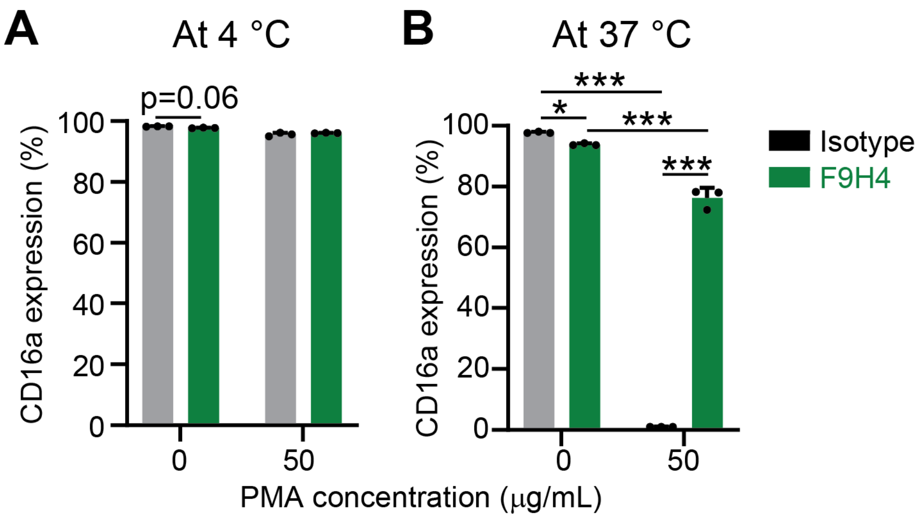

Supplementary Figure 13. CD16a expression levels in NK cells kept cold or warm. NK cells were incubated for 3 hours at the indicated temperatures with the indicated PMA concentrations plus mAbs, followed by flow cytometry analyses. Data are mean ± standard deviation of triplicates, represent three independent experiments, and were analyzed by two-way ANOVA with Bonferroni's test.

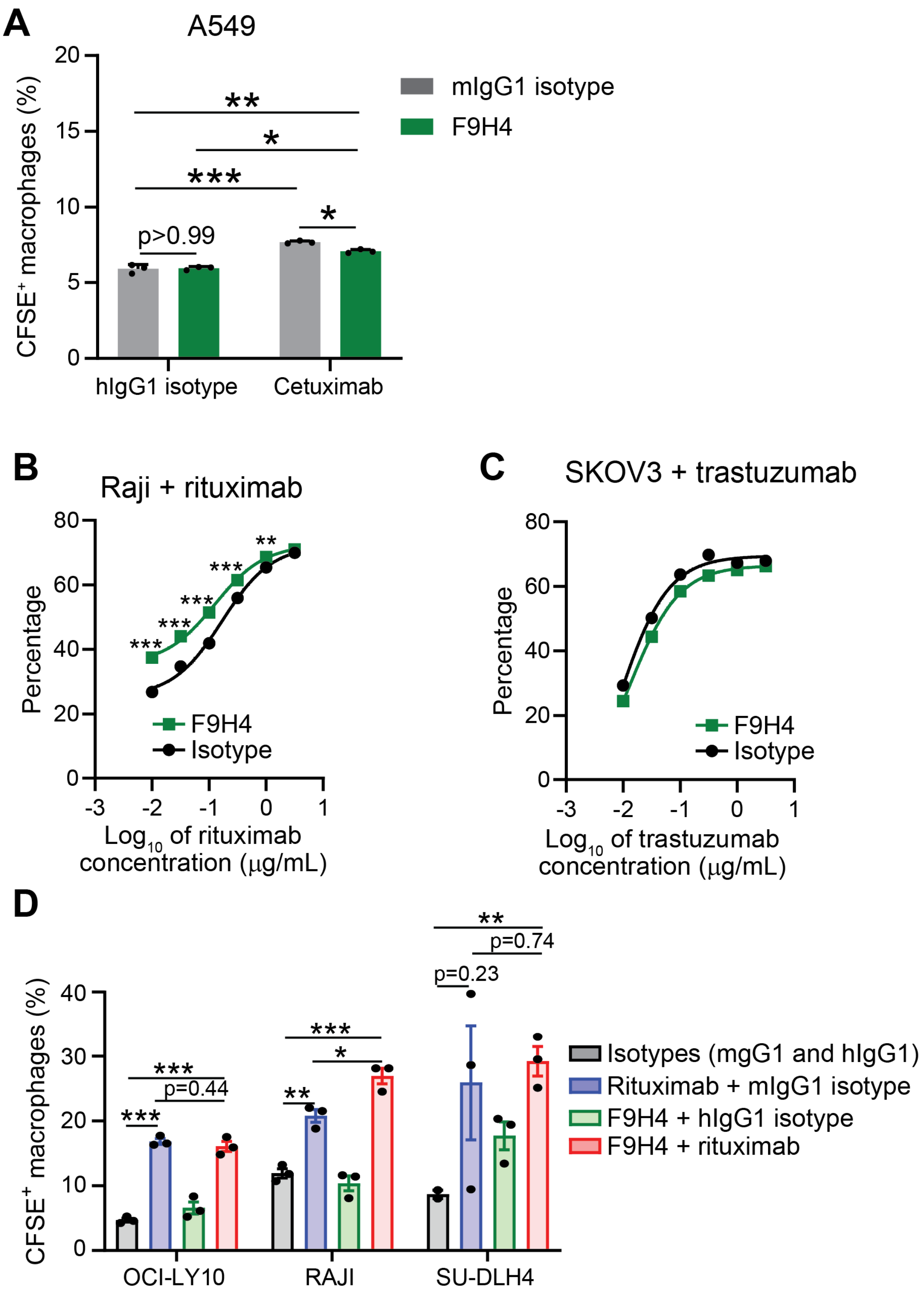

Supplementary Figure 14. Analyses of ADCC and ADCP against tumor cell lines *in vitro*. (A and D) Intermediate and non-classical monocytes (A) or total monocytes (D) were induced to differentiate to macrophages with 7-14 days of GM-CSF and used in 1-hour phagocytosis assays whereby A549 cells (A) or the indicated lymphoma cells (D) were labelled with CFSE and the co-

cultures were treated with the indicated antibodies. Cells were detached by EDTA plus scratching and analyzed by flow cytometry, with macrophage identification with CD11b and CD45. **(B-C)** Human NK cells were isolated by negative selection from the blood of volunteer donors and cultured with interleukin 15 for 3-5 days prior to use as effector cells. These NK cells were co-cultured for 4-hours at 1:1 ratio with the indicated cell lines in the presence of the indicated antibodies, followed by analyses of NK cell degranulation by flow cytometry with classical markers (CD107a, CD56, CD45). Data are mean ± standard deviation (A) or standard error (B, D) of triplicates (A-B and D) except in 'D', SU-DLH4 isotypes (mIgG1 and hIgG1), which is duplicate, or one replicate per data point (C), represent three independent experiments (A-D), and were analyzed by two-way ANOVA with Bonferroni's test (A-B, D). In B, the error bars are too small and cannot be visualized but were included during graph construction. $^{**}p<0.01$, $^{*}p<0.05$.

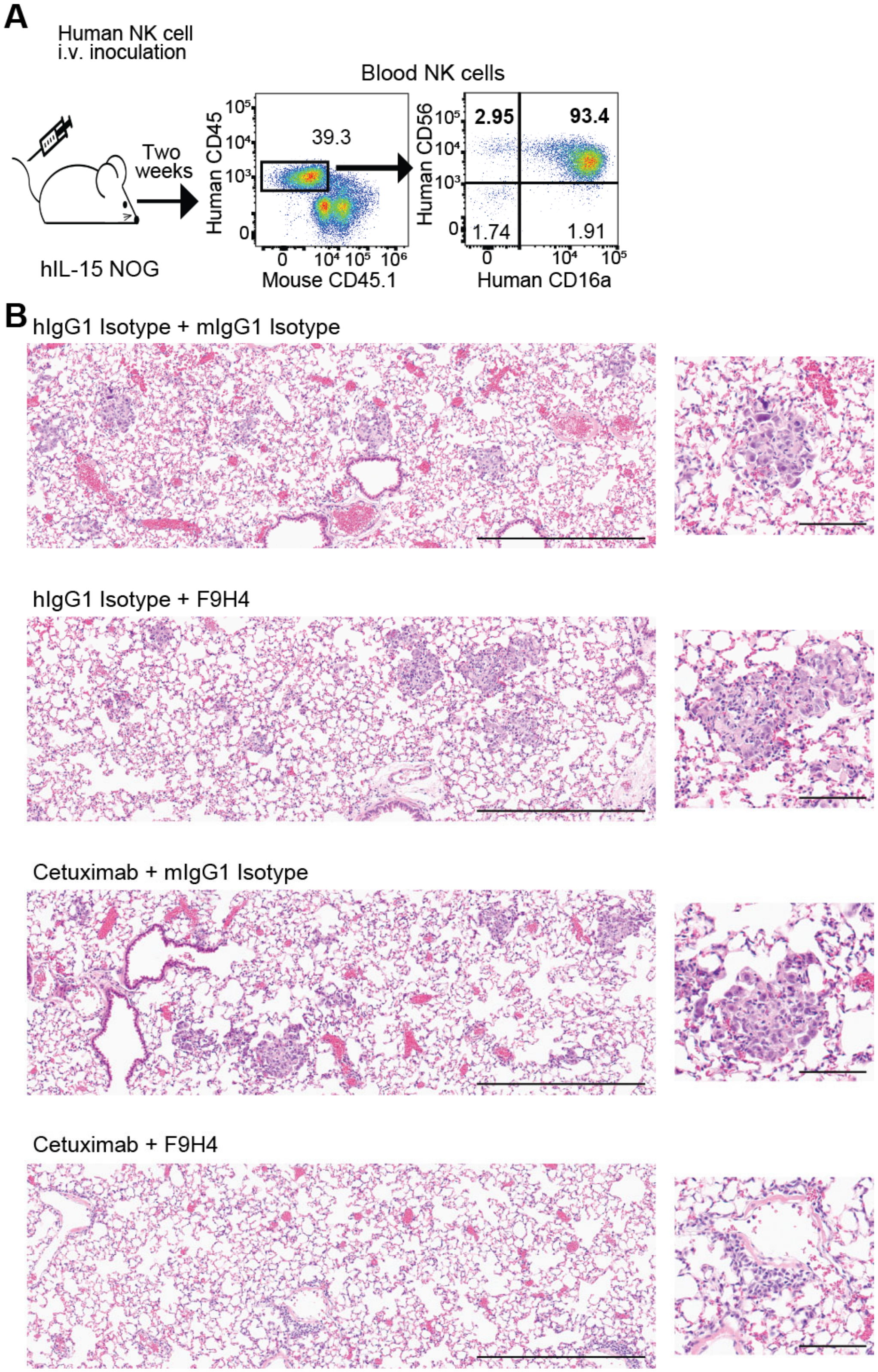

Supplementary Figure 15. F9H4+cetuximab inhibit A549 metastasis formation in hIL-15 NOG mice that were reconstituted with human NK cells. (A) hIL-15 NOG mice were inoculated

intravenously with 1 x 10^6 human primary NK cells and, two weeks after, the NK cells in the blood were analyzed by flow cytometry. Data represent five mice. **(B)** Histopathology that represent the data in Figure 3I. The scale bars in the left images correspond to 500 μm, and the scale bars in the right images correspond to 100 μm.

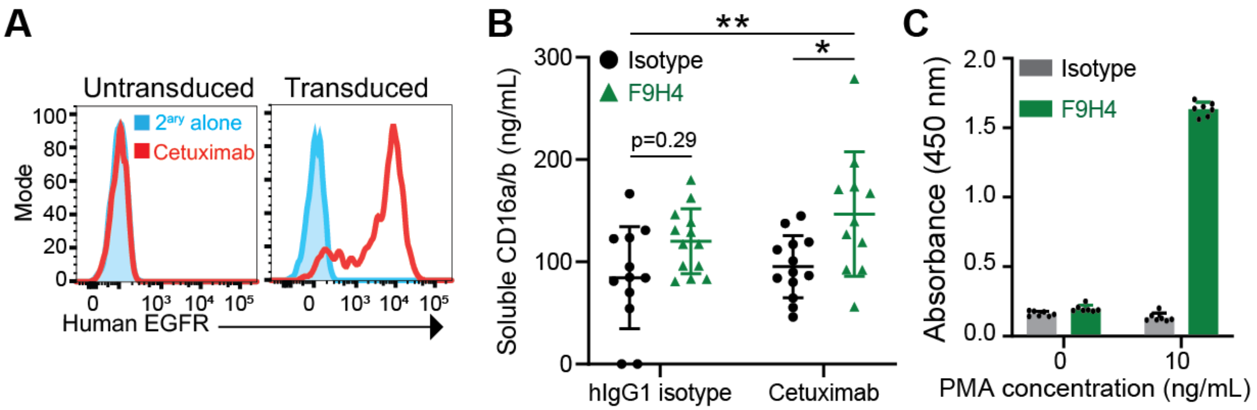

Supplementary Figure 16. The LLC1-hEGFR model in hFcR mice and binding of F9H4 to "cleaved CD16a". (A) Expression of hEGFR in the engineered LLC1 cell line. **(B)** Quantification by ELISA of soluble CD16a/b molecules shed in the sera of hFcR mice. Isotype + hIgG1 isotype n=12, Isotype + cetuximab n=13, F9H4 + hIgG1 isotype n=13, F9H4+cetuximab n=11. **(C)** F9H4 bound cleaved CD16a. MOLM13-CD16a^{WT} cells were treated for one hour as indicated, followed by collection of supernatants that were added to ELISA plates, which were pre-coated with F9H4. This assay uses biotinylated anti-CD16a/b detection antibody. Data represent three (A, C) or are pooled of two (B) independent experiments, are mean ± standard deviation (B, C) of seven replicates per group (C), and were analyzed by two-way ANOVA with Bonferroni's test (B). Each dot represents one mouse (B). **p<0.01, *p<0.05.

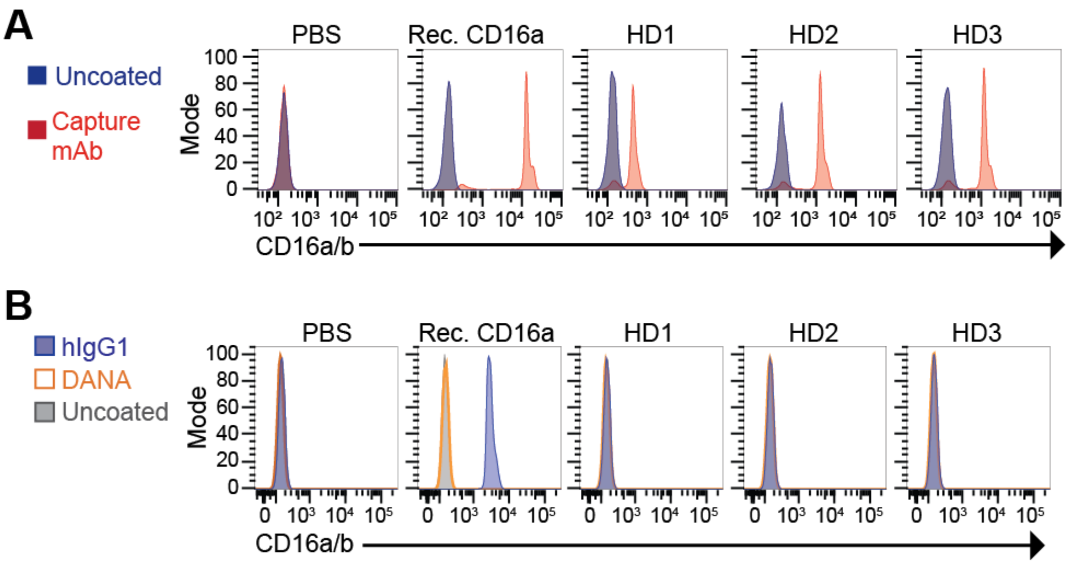

Supplementary Figure 17. Detection of soluble CD16a/b molecules shed into the human plasma. (A) A flow cytometry bead assay that detects soluble CD16a/b in the human plasma from healthy donors (HDs). Flow cytometry beads were covalently linked to anti-CD16a/b capture antibody, incubated with HDs' plasma samples diluted 1:100, followed by incubation with PE-conjugated anti-CD16a/b detection antibody. Rec. CD16a = recombinant CD16a protein, which is a positive control. **(B)** Cleaved CD16a/b in human plasma samples did not bind the Fc. Flow cytometry beads were covalently linked to hIgG1 WT or DANA, which serves as a negative control, and incubated with recombinant CD16a, which is a positive control, or human plasma samples. The binding is detected by PE-conjugated anti-CD16a/b detection antibody. Data represent three (B) or two (A) independent experiments. Data with three HDs represent nine HDs (A-B).

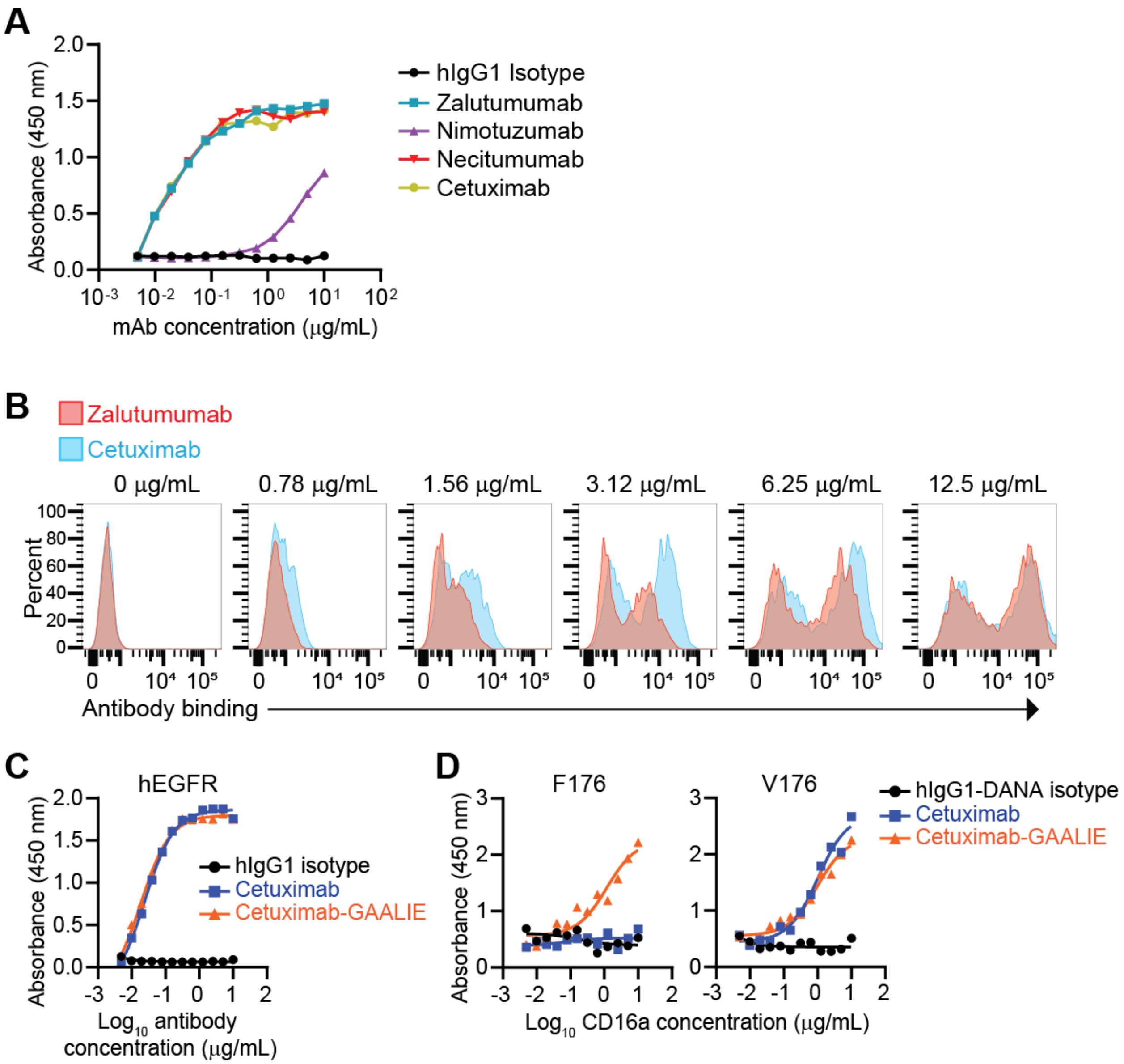

Supplementary Figure 18. Validation of EGFR mAbs. (A) ELISA for the binding of the indicated mAbs to recombinant human EGFR protein. **(B)** LLC1-hEGFR cells were incubated with the indicated concentrations of cetuximab or zalutumumab, which was followed by incubation with APC-conjugated anti-human IgG monoclonal antibody and analyses by flow cytometry. **(C)** Cetuximab-GAALIE binds hEGFR to a similar extent compared to cetuximab. Standard ELISA for the reactivity of the indicated antibodies to hEGFR. **(D)** Cetuximab-GAALIE binds CD16a-F176 with higher affinity compared to the binding by cetuximab, whereby both antibodies display similar binding affinity to CD16a-V176. The indicated antibodies were coated in wells of ELISA plates, followed by bovine serum albumin-mediated blockade and addition on the indicated polymorphic variants of biotinylated CD16a. The reaction was revealed with peroxidase-conjugated streptavidin and TMB. Data are one replicate per data point (A-D), represent three independent experiments (A-D), and were analyzed by non-linear regression (A, C-D).

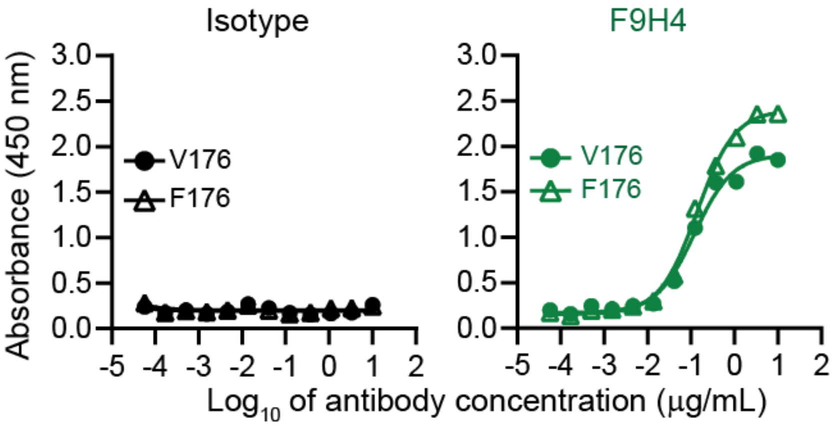

Supplementary Figure 19. F9H4 binds to both polymorphic variants of CD16a. ELISA for the reactivity of the indicated antibodies against the indicated recombinant proteins. Data are one replicate per data point and represent three independent experiments.

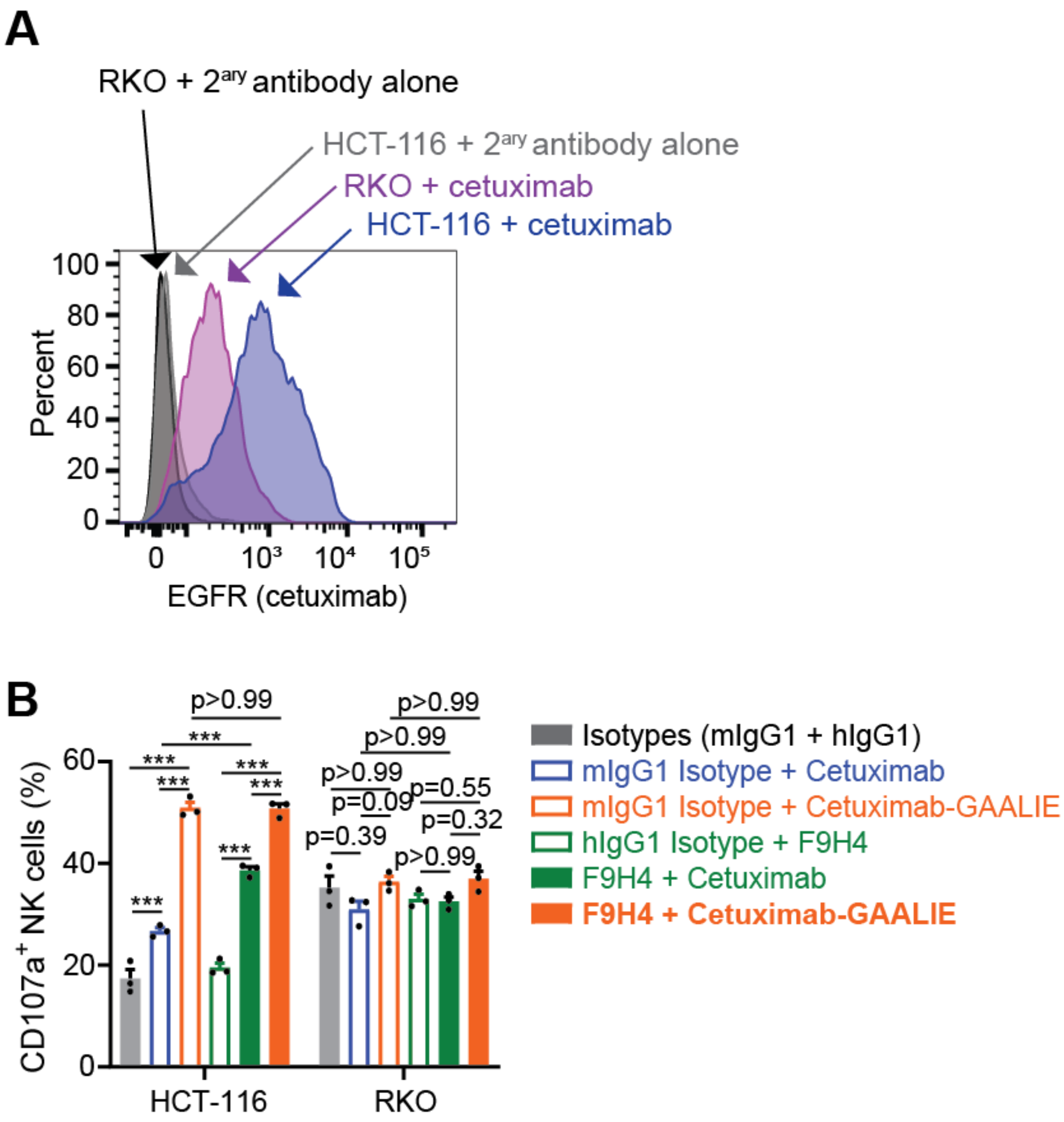

Supplementary Figure 20. EGFR expression in, and NK cell degranulation against colorectal cancer cell lines. (A) Binding of cetuximab to the indicated human colorectal cancer cell lines. Analyses by flow cytometry. **(B)** NK cell CD107a externalization assays against the indicated cell lines. Data represent two (A) or three (B) independent experiments, are one replicate per group (A) or mean + standard error of triplicates (B), and were analyzed by two-way ANOVA with Bonferroni's test (B). ***p<0.001.

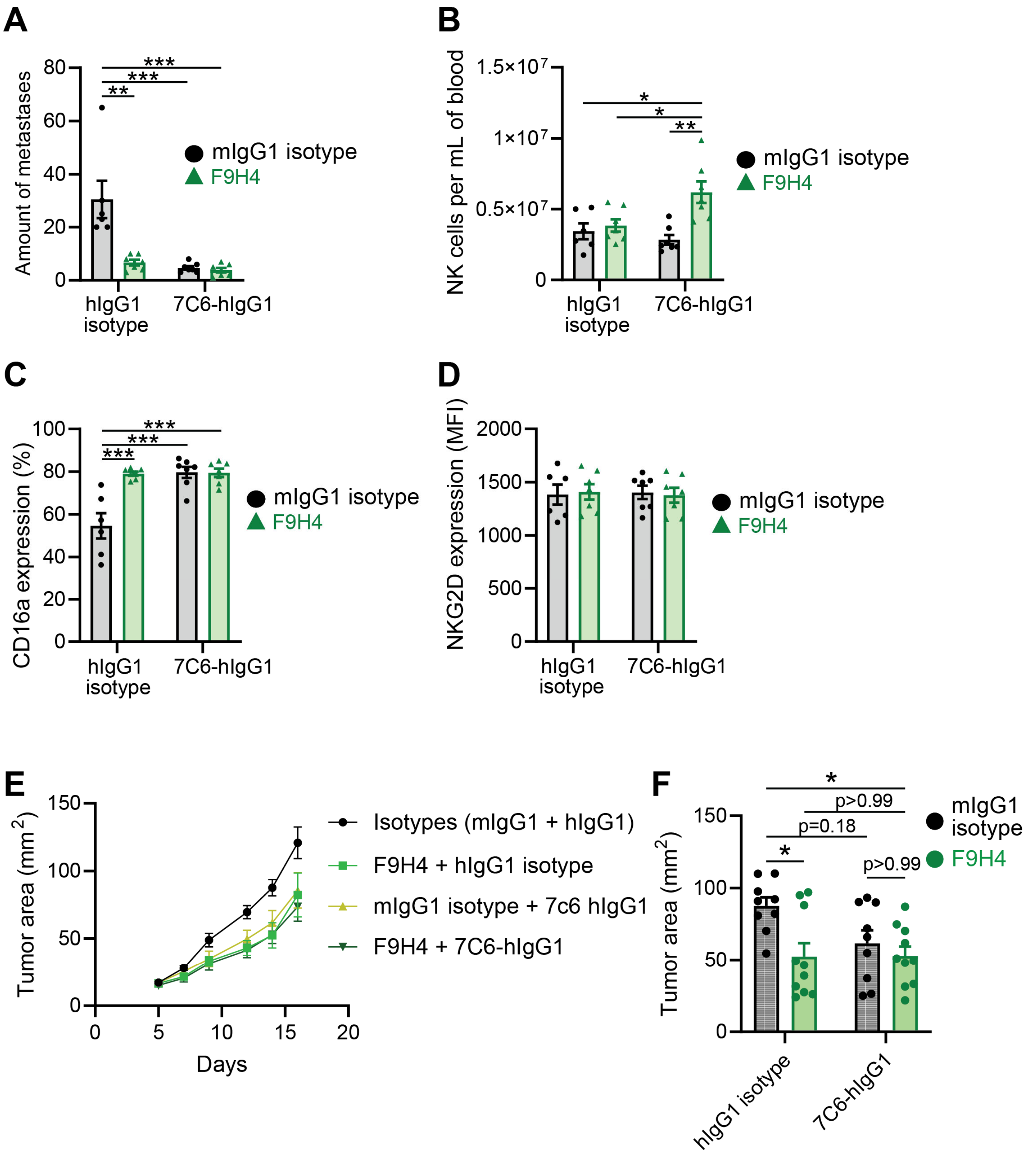

Supplementary Figure 21. B16F10-MICA metastasis and LLC1-MICA tumor models in hFcR mice and treatment with mAbs. (A-D) hFcR mice were inoculated intravenously with B16F10-MICA cells and treated with 0.2 mg 7C6-hIgG1 or isotype plus 0.1 mg F9H4 or isotype on days 1, 2, and 7 relative. Analyses were done on day 14. **(A)** Quantification of the number of metastases in the lungs by stereomicroscopy. hIgG1 isotype + mIgG1 isotype n=6, hIgG1 isotype + F9H4 n=7, 7C6-hIgG1 + isotype n=7, 7C6-hIgG1 + F9H4 n=7. **(B-D)** Analyses of blood NK cells by flow cytometry. hIgG1 isotype + mIgG1 isotype n=6, hIgG1 isotype + F9H4 n=7, 7C6-hIgG1 +

isotype n=7, 7C6-hIgG1 + F9H4 n=7. **(B)** Absolute numbers of NK cells per mL of blood. **(C-D)** Surface CD16a (C) and NKG2D (D) expressions. **(E-F)** hFcR mice were inoculated subcutaneously with LLC1-MICA cells and treated with 0.2 mg 7C6-hIgG1 or isotype plus 0.1 mg F9H4 or isotype on days 5 and 6 and once per week. Tumor were measured with digital caliper. hIgG1 isotype + mIgG1 isotype n=9, hIgG1 isotype + F9H4 n=10, 7C6-hIgG1 + isotype n=9, 7C6-hIgG1 + F9H4 n=10. **(F)** Tumor areas on day 14. Data are mean ± standard error (A-F) and pooled of two independent experiments (A-F). Each dot represents one mouse (A-D, F). Statistical analyses by two-way ANOVA with Bonferroni's test. *p<0.05, **p<0.01, and ***p<0.001.

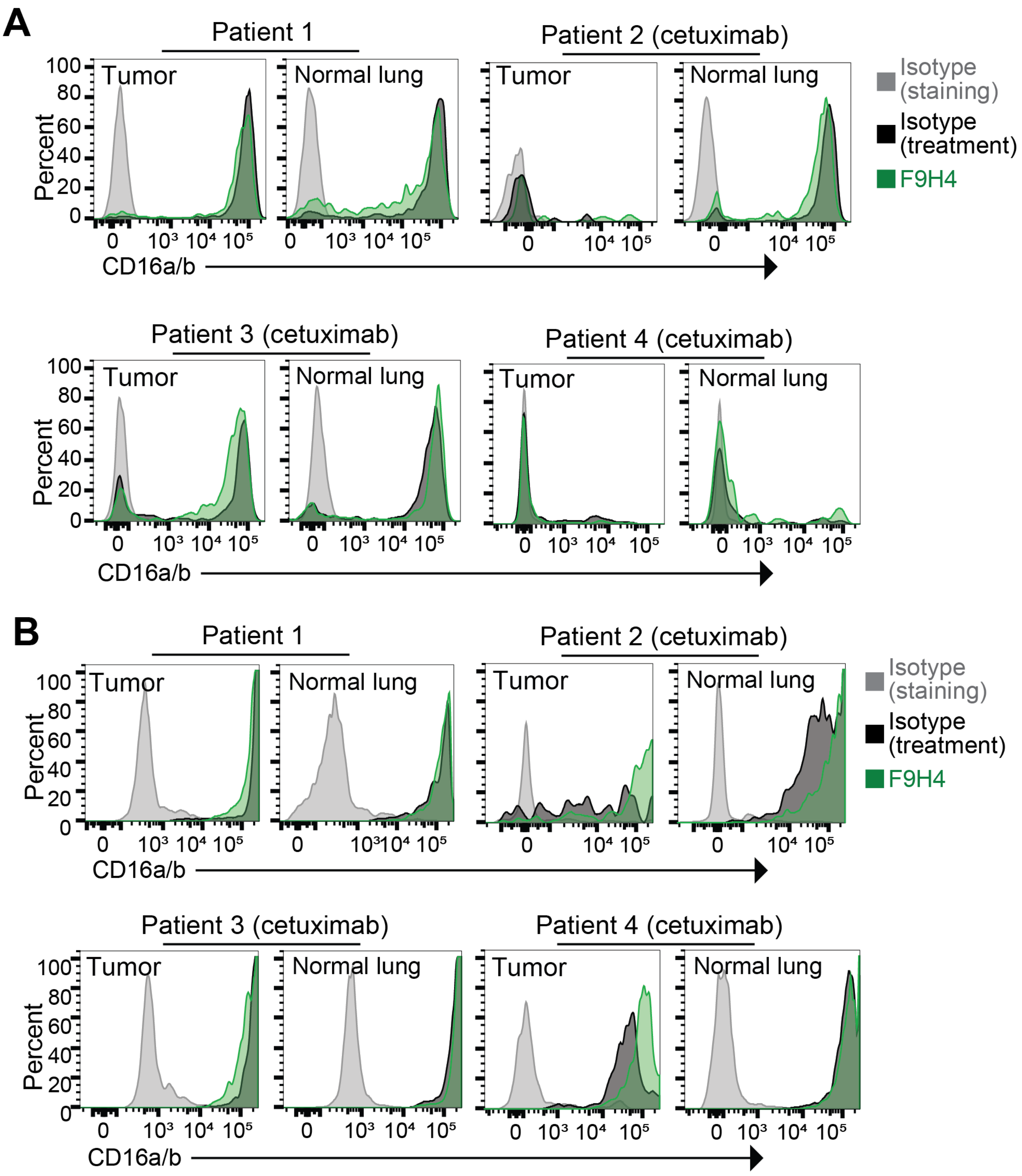

Supplementary Figure 22. Analyses of surface CD16a/b in NK cells and neutrophils from the tissue explants. Data that complement the data shown in Figure 5G. **(A-B)** Expression of CD16a/b in NK cells (A) and neutrophils (B). **(A)** NK cells were identified as $CD45^{+}CD56^{+}CD3^{-}$ cells. **(B)** Neutrophils were identified as $CD45^{+}CD15^{+}$ cells. A total of four patients were analized and are all shown here (A-B).

Supplementary Table 1

Name	Clone	Catalog Number	Brand	Dilution
Anti-mouse mIgG1	RMG1-1	406604	Biolegend	1 ug/mL
Anti-mouse IgG2a	RMG2a-62	407104	Biolegend	1 ug/mL
Anti-mouse IgG2b	RMG2b-1	406704	Biolegend	1 ug/mL
Anti-mouse IgG3	RMG3-1	406803	Biolegend	1 ug/mL
Anti-human IgG1 Fc	M1310G05	410718	Biolegend	1 ug/mL
Anti-mouse mIgG1	MOPC-21	400197	Biolegend	100 ug/mL
Anti-human IgG Fc	M1310G05	410711	Biolegend	1/150
Anti-human CD69	FN50	310916	Biolegend	1/150
Anti-human CD14	63D3	367120	Biolegend	1/150
Anti-CD107a	H4A3	328610	Biolegend	1/150
Anti-mouse CD45.1	A20	110730	Biolegend	1/150
Anti-human CD45	2D1	368518	Biolegend	1/150
Anti-mouse CD45.2	104	109814	Biolegend	1/150
Anti-mouse CD49b	HMα2	103518	Biolegend	1/150
Anti-mouse CD3e	17A2	100216	Biolegend	1/150
Anti-human CD3	OKT3	317344	Biolegend	1/150
Anti-mouse NK1.1	PK136	108749	Biolegend	1/200
Anti-human CD15	W6D3	323012	Biolegend	1/150
Anti-human CD11b	M1/70	101210	Biolegend	1/150
Anti-human CD56	HCD56	318314	Biolegend	1/150
Anti-human CD64	S18012C	399506	Biolegend	1/150
Anti-human CD16	3G8	302056	Biolegend	1/150
Anti-human CD33	P67.6	366623	Biolegend	1/150
Anti-human interferon-gamma	4S.B3	502509	Biolegend	1/150
Mouse IgG2b isotype	MPC-11	400369	Biolegend	10 ug/mL
Mouse IgG3 isotype	MG3-35	401301	Biolegend	10 ug/mL
Anti-human EGFR	Cetuximab	ENZABS4860200	Enzo Life Sciences	1 ug/mL
Anti-human HER2	Trastuzumab	ENZABS4900200	Enzo Life Sciences	1 ug/mL
Anti-mouse IgG	NA	BAF018	R&D Systems	0.2 mg/mL
Anti-mouse IgG Fab and Fab2	NA	315-005-006	Jackson ImmunoResearch Laboratories	1 ug/mL
Cetuximab-gaalie	Cetuximab	ICH4004Fc	Ichorbio	10 ug/mL
Zalatumumab	Zalatumumab	ICH5116	Ichorbio	10 ug/mL
Nimotuzumab	Nimotuzumab	ICH4008	Ichorbio	10 ug/mL
Necitumumab	Necitumumab	ICH5121	Ichorbio	10 ug/mL
Human IgG1 isotype	NA	BE0297	Bio X Cell	10 ug/mL
Mouse IgG1 isotype	MOPC21	BE0083	Bio X Cell	10 ug/mL
Mouse IgG2a isotype	C1184	BE0085	Bio X Cell	10 ug/mL

Anti-human CD16	3G8	302002	Biolegend	10 ug/mL
