# Supplementary material for: A monoclonal antibody that inhibits the shedding of CD16a and CD16b and promotes antibody-dependent cellular cytotoxicity against tumors
Source: Nat Commun. 2025 Nov 11;16:9915. doi: 10.1038/s41467-025-64862-5 (PMC12606087; doi:10.1038/s41467-025-64862-5)
Supplement: Supplementary file 2 — Reporting Summary [file 41467_2025_64862_MOESM2_ESM.pdf]

nature portfolio

Corresponding author(s): Lucas Ferrari de Andrade

Last updated by author(s): Sep 17, 2025

# Reporting Summary

Nature Portfolio wishes to improve the reproducibility of the work that we publish. This form provides structure for consistency and transparency in reporting. For further information on Nature Portfolio policies, see our Editorial Policies and the Editorial Policy Checklist.

## Statistics

For all statistical analyses, confirm that the following items are present in the figure legend, table legend, main text, or Methods section.

| n/a | Confirmed | |
|---|---|---|
| ☐ | ☒ | The exact sample size (*n*) for each experimental group/condition, given as a discrete number and unit of measurement |
| ☐ | ☒ | A statement on whether measurements were taken from distinct samples or whether the same sample was measured repeatedly |
| ☐ | ☒ | The statistical test(s) used AND whether they are one- or two-sided<br>*Only common tests should be described solely by name; describe more complex techniques in the Methods section.* |
| ☒ | ☐ | A description of all covariates tested |
| ☒ | ☐ | A description of any assumptions or corrections, such as tests of normality and adjustment for multiple comparisons |
| ☐ | ☒ | A full description of the statistical parameters including central tendency (e.g. means) or other basic estimates (e.g. regression coefficient) AND variation (e.g. standard deviation) or associated estimates of uncertainty (e.g. confidence intervals) |
| ☐ | ☒ | For null hypothesis testing, the test statistic (e.g. *F*, *t*, *r*) with confidence intervals, effect sizes, degrees of freedom and *P* value noted<br>*Give P values as exact values whenever suitable.* |
| ☒ | ☐ | For Bayesian analysis, information on the choice of priors and Markov chain Monte Carlo settings |
| ☒ | ☐ | For hierarchical and complex designs, identification of the appropriate level for tests and full reporting of outcomes |
| ☒ | ☐ | Estimates of effect sizes (e.g. Cohen's *d*, Pearson's *r*), indicating how they were calculated |

*Our web collection on statistics for biologists contains articles on many of the points above.*

## Software and code

Policy information about availability of computer code

| | |
|---|---|
| Data collection | No public data was collected or used in this study. |
| Data analysis | No public data was collected or used in this study. |

For manuscripts utilizing custom algorithms or software that are central to the research but not yet described in published literature, software must be made available to editors and reviewers. We strongly encourage code deposition in a community repository (e.g. GitHub). See the Nature Portfolio guidelines for submitting code & software for further information.

## Data

Policy information about availability of data

All manuscripts must include a data availability statement. This statement should provide the following information, where applicable:

- Accession codes, unique identifiers, or web links for publicly available datasets
- A description of any restrictions on data availability
- For clinical datasets or third party data, please ensure that the statement adheres to our policy

All data can be provided upon requests made directly to the corresponding author.

## Research involving human participants, their data, or biological material

Policy information about studies with human participants or human data. See also policy information about sex, gender (identity/presentation), and sexual orientation and race, ethnicity and racism.

| | |
|---|---|
| Reporting on sex and gender | This was not human subject research. The samples utililzed were provided in a de-identified manner. |
| Reporting on race, ethnicity, or other socially relevant groupings | This was not human subject research. The samples utililzed were provided in a de-identified manner. |
| Population characteristics | This was not human subject research. |
| Recruitment | This was not human subject research. The blood samples were from volunteers that donated blood to the New York BLood Center, and the tumor and lung specimens were from patients that received the standard of care at the Mount Sinai Hospital. |
| Ethics oversight | The collection, storage, and distribution of these patient-derived materials by the Biorepository and Pathology Core (Mount Sinai) were approved by the Institutional Review Board (Mount Sinai), and the use of these specimens in the experiments were approved by the Lung Cancer Tissue Utilization Committee (Mount Sinai). |

Note that full information on the approval of the study protocol must also be provided in the manuscript.

# Field-specific reporting

Please select the one below that is the best fit for your research. If you are not sure, read the appropriate sections before making your selection.

☒ Life sciences ☐ Behavioural & social sciences ☐ Ecological, evolutionary & environmental sciences

For a reference copy of the document with all sections, see nature.com/documents/nr-reporting-summary-flat.pdf

# Life sciences study design

All studies must disclose on these points even when the disclosure is negative.

| | |
|---|---|
| Sample size | In vitro experiments were done with at least triplicate to account for technical variability. In vivo experiments were done with at least five mice per group to account for inter individual variability. |
| Data exclusions | No data were excluded from the analyses. |
| Replication | In vitro experiments were repeated at least three times, except Figure 2C that was done twice. In vivo experiments were done at least twice. Replications were successful. |
| Randomization | Allocation was random. |
| Blinding | The investigators were not blinded to the experimental groups, because the analyses were objective. |

# Reporting for specific materials, systems and methods

We require information from authors about some types of materials, experimental systems and methods used in many studies. Here, indicate whether each material, system or method listed is relevant to your study. If you are not sure if a list item applies to your research, read the appropriate section before selecting a response.

## Materials & experimental systems

| n/a | Involved in the study | |
|---|---|---|
| ☐ | ☒ | Antibodies |
| ☐ | ☒ | Eukaryotic cell lines |
| ☒ | ☐ | Palaeontology and archaeology |
| ☐ | ☒ | Animals and other organisms |
| ☒ | ☐ | Clinical data |
| ☒ | ☐ | Dual use research of concern |
| ☒ | ☐ | Plants |

## Methods

| n/a | Involved in the study | |
|---|---|---|
| ☒ | ☐ | ChIP-seq |
| ☐ | ☒ | Flow cytometry |
| ☒ | ☐ | MRI-based neuroimaging |

## Antibodies

| | |
|---|---|
| Antibodies used | F9H4 was generated by our laboratory via hybridoma technology, and the recombinant version was produced in 293 T cells. Cetuximab, Cetuximab-GAALIE, Necitumumab, Nimotuzumab, and Zalutumumab were from IchorBio. Rituximab and trastuzumab were from Enzo Life Sciences. The Methods section include details about each antibody that was used by flow cytometry. |
| Validation | F9H4, cetuximab, and cetuximab-GAALIE were validated by ELISA as shown in the manuscript (Figure 1A, Supplementary Figure 6A-B and 12A-B). Nimotuzumab, necitumumab, and zalutumumab were validated by ELISA (Figure S18A). We did not validate rituximab or trastuzumab. |

## Eukaryotic cell lines

Policy information about cell lines and Sex and Gender in Research

| | |
|---|---|
| Cell line source(s) | All cell lines were from ATCC, except KPN1.1 that was a kind gift from Nikhil Joshi (Yale School of Medicine) and MOLM13 that was a kind gift from Thomas Pabst (University of Bern). |
| Authentication | The ATCC cell lines were not authenticated because they are from ATCC and authentication would be done by ATCC. The EGFR, CD16a, and MICA engineered cell lines were authenticated by flow cytometry. |
| Mycoplasma contamination | All cell lines were tested negative for mycoplasma. |
| Commonly misidentified lines (See ICLAC register) | We did not use such cell lines. |

## Animals and other research organisms

Policy information about studies involving animals; ARRIVE guidelines recommended for reporting animal research, and Sex and Gender in Research

| | |
|---|---|
| Laboratory animals | Fc gamma receptor-humanized mice and hIL-15 NOG mice. |
| Wild animals | We did not use wild animals. |
| Reporting on sex | Most of the experiments were done in male mice because we established that male mice develop larger tumors. |
| Field-collected samples | We did not collect field samples. |
| Ethics oversight | The study was approved by the Institutional Animal Care and Use Committee in the Icahn School of Medicine at Mount Sinai |

Note that full information on the approval of the study protocol must also be provided in the manuscript.

## Plants

| | |
|---|---|
| Seed stocks | We did not use plants. |
| Novel plant genotypes | We did not use plants. |
| Authentication | We did not use plants. |

## Flow Cytometry

### Plots

Confirm that:

- [ ] The axis labels state the marker and fluorochrome used (e.g. CD4-FITC).
- [x] The axis scales are clearly visible. Include numbers along axes only for bottom left plot of group (a 'group' is an analysis of identical markers).
- [x] All plots are contour plots with outliers or pseudocolor plots.
- [x] A numerical value for number of cells or percentage (with statistics) is provided.

## Methodology

| | |
|---|---|
| Sample preparation | Please refer to the Methods section. |
| Instrument | LSRII Fortessa from BD. |
| Software | FACS Diva and FlowJo. |
| Cell population abundance | This study does not identify a new cell population by flow cytometry. The methods used are standard. |
| Gating strategy | We started with FSC/SSC gatings, followed by FSCA/FSCH, SSCA/SSCH, and dead cell removal gatings. |

☐ Tick this box to confirm that a figure exemplifying the gating strategy is provided in the Supplementary Information.
